# Predictors of Healthcare Service Utilization for Mental Health Reasons

**DOI:** 10.3390/ijerph111010559

**Published:** 2014-10-15

**Authors:** Marie-Josée Fleury, André Ngamini Ngui, Jean-Marie Bamvita, Guy Grenier, Jean Caron

**Affiliations:** 1 Department of Psychiatry, McGill University, Montreal, QC H3A 0G4, Canada; 2 Montreal Addiction Rehabilitation Centre—University Institute (CRDM-IU), Montreal, QC H2M 2E8 Canada; E-Mail: ngui@justice.com; 3 Psychosocial Division, Douglas Hospital Research Centre, Montreal, QC H4H 1R3, Canada; E-Mails: jean-marie.bamvita@douglas.mcgill.ca (J.-M.B.); guy.grenier@douglas.mcgill.ca (G.G.); jean.caron@douglas.mcgill.ca (J.C.)

**Keywords:** mental health, service utilization, Andersen behavioral model, longitudinal study, catchment area research

## Abstract

This study was designed to identify: (1) predictors of 12-month healthcare service utilization for mental health reasons, framed by the Andersen model, among a population cohort in an epidemiological catchment area; and (2) correlates associated with healthcare service utilization for mental health reasons among individuals with and without mental disorders respectively. Analyses comprised univariate, bivariate, and multiple regression analyses. Being male, having poor quality of life, possessing better self-perception of physical health, and suffering from major depressive episodes, panic disorder, social phobia, and emotional problems predicted healthcare service utilization for mental health reasons. Among individuals with mental disorders, needs factors (psychological distress, impulsiveness, emotional problems, victim of violence, and aggressive behavior) and visits to healthcare professionals were associated with healthcare service utilization for mental health reasons. Among individuals without mental disorders, healthcare service utilization for mental health reasons is strongly associated with enabling factors such as social support, income, environmental variables, and self-perception of the neighborhood. Interventions facilitating social cohesion and social solidarity in neighborhood settings may reduce the need to seek help among individuals without mental disorders. Furthermore, in their capacity as frontline professionals, general practitioners should be more sensitive in preventing, detecting, and treating mental disorders in routine primary care.

## 1. Introduction

According to the World Health Organization, mental disorders are the world’s leading cause of disability after cardiovascular disease [1,2]. According to a recent study, mental disorders account for approximately 33% of time lost to disability worldwide [3]. Excluding neurological conditions affecting the brain, the rates of lifetime prevalence of mental disorders among adults worldwide ranges from 12.2% to 48.6%, and 12-month prevalence from 8.4% to 29.1% [4]. In the United States, community epidemiological surveys estimate that about 30% of the adult population meet “the criteria for a 12-month mental disorder” [5]. As for the 2012 Canadian Community Health Survey among Canadians aged 15 years or more, 10% experienced at least one mental disorder in the previous 12 months [6]. In the province of Quebec, the estimated prevalence of mental disorders was 12% in the general population in 2009–2010 [7]. 

Mental disorders are associated with major social and economic consequences. Patients with mental disorders have high mortality rates [8], poor quality of life [9], lower self-esteem [10], and lack educational and income-generating opportunities, thus limiting their chances of economic development and depriving them of social networks and status within the community [11]. They also experience a variety of chronic physical health problems such as hypertensive and cerebrovascular diseases [12]. Among individuals with major depression in a 12-month period, 66% are also affected by chronic physical disease [13]. According to a WHO survey, 52% of individuals with heart diseases also experience symptoms of depression and 30% meet the diagnostic criteria for major depression [14]. 

Despite the pervasive need for mental health treatment among individuals with mental disorders, it is generally acknowledged that a great proportion of them do not use healthcare services [15,16,17,18,19]. Canadian studies report that less than 40% of Canadians suffering from mental disorders consult a healthcare professional or services for mental health reasons [20,21]. Thus, there is a pressing need to identify factors that foster healthcare service utilization for mental health reasons. The decision to seek help for mental disorders is a complex process that involves personal socio-demographic characteristics, culturally mediated interpretations of symptoms, availability of healthcare services, economic and socio-structural factors, and healthcare service organization [22]. A number of healthcare service utilization models exist [23,24,25,26,27,28,29], but most studies of healthcare service utilization for mental health reasons have used the behavioral model developed by Andersen in the 1960s [15,16,17,18,30,31,32]. This model conceptualized healthcare service utilization as a function of individuals’ predisposing, enabling, and needs characteristics [33,34].

The predisposing factors center on individual characteristics prior to an illness episode (for example, age, gender, life satisfaction, marital status, self-rated health). Studies have revealed that a high level of education [35], being a woman [36] and young [37] were variables correlated with seeking help for mental disorders. The enabling factors centers on the idea that variables such as income, community and system resources (including social support and service availability and accessibility) are main determinants of healthcare service utilization. A study using an administrative database found that in central Toronto, lower socioeconomic status was associated with a lesser likelihood of consulting a psychiatrist or a general practitioner for mental health reasons [38]. Previous studies have found a positive correlation between good attitude toward health care providers [39], good perceived social support [40] or social ties [41,42], high income [43,44,45] and healthcare service utilization for mental health reasons. Needs factors refer mainly to the type and number of symptoms and the degree of severity of an illness. Previous studies have shown that a diagnosis of mental disorder and the severity and duration of mental health symptoms were associated with seeking help for mental health reasons [46,47]. Numerous authors have also reported that patients with co-occurring disorders are more likely to use healthcare service utilization for mental health reasons [48,49,50]. Most researchers using the behavioral model concluded that needs were the main correlates of healthcare service utilization [51,52]. The relative importance of the contribution of each component, however, varies by type of healthcare services used [53,54,55].

In addition, most studies have focused on cross-sectional population surveys [56] or on patients recruited in hospital settings (clinical sample) [57]. Few have assessed predictors associated with healthcare service utilization for mental health reasons in a catchment area, using a comprehensive framework [58,59]. Moreover, several factors such as environmental variables and self-perception of the neighborhood, which may be predictors of healthcare service utilization for mental health reasons, have received little or no attention in the literature.

Furthermore, most epidemiological studies have found that nearly 50% of individuals who use healthcare services for mental health reasons do not have a diagnosis of mental disorder in the 12 months before the interview [60,61,62,63,64]. This situation may be the result of overuse or misuse of healthcare services [60]. Some individuals may be affected by psychological distress or emotional problems resulting from bereavement, divorce, financial problems or other stressful events. Others may have previously been affected by a mental disorder and consulted a professional to determine if they had suffered a relapse. Accordingly, it is possible that individuals without mental disorders use specialized mental healthcare services rather than primary care. Better knowledge of factors distinguishing users of services for mental health reasons with and without mental disorders would be useful in improving the distribution of healthcare services.

The purpose of this study was to: (1) assess longitudinal predictors of 12-month healthcare service utilization for mental health reasons among a population cohort in an epidemiological catchment area, framed by the Andersen model; and (2) identify variables associated with healthcare service utilization for mental health reasons among individuals with and without mental disorders, respectively.

## 2. Methods 

### 2.1. Study Design and Setting

Our research focused on an epidemiological catchment area located in southwestern Montreal. Greater Montreal is Canada’s second-largest urban center, with a population of 3.6 million. The catchment area is home to 269,720 people and includes four neighborhoods, which range in population from 29,680 to 72,420. Immigrants in the catchment area represent 17% of the population (*vs*. 26% in Montreal). The proportion of low-income households is 36% (*vs*. 23% in the province of Quebec and 35% in Montreal). Low-income households are located mainly in two of the four neighborhoods where close to half of the residents are low-income earners. The catchment area included a diversity of services, mainly in healthcare and mental healthcare. The latter services and the socioeconomic characteristics of the area are described in detail in other publications [17,65].

### 2.2. Selection Criteria and Survey Sample

For inclusion in the survey, participants had to be aged between 15 and 65 and reside in the study catchment area. The objective was to obtain a representative sample of the target population, both geographically (that is, recruiting participants from all areas of the territory) and proportionally to population density and socio-economic status (representative of educational level). Data were collected by specially trained interviewers on two separate occasions at a two-year interval.

At T1 (June 2007 to December 2008), 2434 individuals were randomly selected for the survey. On average, 600 individuals were selected in each area: Saint-Henri/Pointe-St-Charles: 612; Lachine/Dorval: 603; Lasalle: 584; Verdun: 635. The mean age of the sample was 42.4 (SD: 13.3). Sixty-three percent were female. Forty-five percent were married or common-law spouses *vs.* 17% divorced or separated and 37% single. Seventy-two percent had post-secondary education and 77% held a job in the last 12 months. French was the first language for 54% of participants and English for 22%. Eighty-two percent were Caucasian. Twenty-four percent of participants were non-European immigrants. Average personal income was CA$28,688 (SD: 31,061) and average household income was CA$49,566 (SD: 51,057).

**Figure 1:**
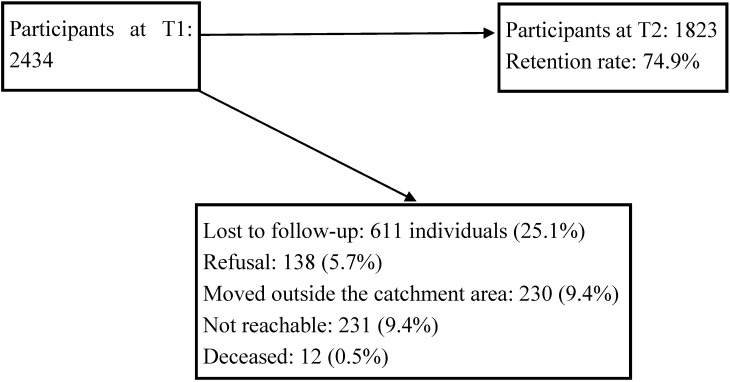
Flowchart of the sample from baseline (T1) to second survey (T2).

All of the participants were contacted for a second interview (T2) from June 2009 to December 2010. Only 611 were lost during the follow-up, for a retention rate of 74.9%, that is, 1823 participants (Figure 1). The attrition rate at T2 (25.1%) included only 138 (5.7%) who refused to participate, 230 (9.4%) who had moved outside the catchment area, 231 (9.4%) who could not be reached, and 12 (0.5%) who had died. This attrition rate after two years was lower than that observed in the American epidemiological catchment areas [66] after one year (20.4%, including 12.6% refusals). The attrition rate was higher among youths, singles, individuals with poorer education, those with lower individual income, and those with substance dependence, which is similar to characteristics previously identified in other epidemiological catchment area studies [67,68,69,70]. The research was approved by the relevant ethics boards. The sampling strategy and data collection (especially at T1) are described in detail in related publications [58,65].

### 2.3. Variables and Measuring Instruments

The dependent variable was “12-month healthcare service utilization for mental health reasons prior to the interview” (yes/no). Healthcare service utilization was measured with the Canadian Community Health Survey questionnaire [71]. It included: psychiatrists, general practitioners, psychologists, social workers, nurses and other health professionals, hospitalizations, rehabilitation centers, and community-based organization services.

Independent variables were measured at baseline (T1) and organized according to the Andersen behavioral model of healthcare service utilization, comprising predisposing factors, enabling factors, needs factors, and type of healthcare service professionals’ utilization (*i.e.*, the practitioners listed in the paragraph above). Predisposing factors included socio-demographic variables (age, gender, education level, problems with the law in the past 12 months, lifelong of problems with the law), and beliefs (self-perception of physical and mental health, satisfaction with life, importance attributed to spirituality, number of children in the household). Enabling factors included source of income, household and personal income, quality of life, self-perception of the neighborhood (physical conditions of the neighborhood score, security score, community involvement scale score, sense of collective efficacy score, resident disempowerment scale score, neighboring behavior scale score), social support and environmental variables (driving distance to the neighborhood community health center, proportion of immigrant population in the neighborhood, mean household income in the neighborhood before income tax). Needs factors included having an mental disorder, number and types of mental disorders (major depression, mania, panic disorder, social phobia, agoraphobia, post-traumatic syndrome disorder (PTSD), alcohol dependence, and drug dependence), having emotional problems, being a victim of violence, and exhibiting aggressive behaviors (all in the past 12 months) and psychological distress score. Variables assessed in this study were measured with the instruments listed in Table 1.

**Table 1:** Measuring Instruments.

Factors	No.	Name	Description
**Predisposing factors**	1	CCHS 1.2 [71]	Canadian Community Health Survey (CCHS): survey questionnaire for socio-demographic characteristics
	2	Satisfaction with Life Domains Scale (SLDS) [72]	20 items organized in 5 domains: daily living and social relationships, living environment, autonomy, intimate relationships, and leisure
**Enabling factors**	3	Sense of Community Scale (SCS) [73]	8 items
	4	Community Participation Scale (CPS) [74]	6 items Measures association between crime victimization, social organization, and participation in neighborhood organization
	5	Resident Disempowerment Scale (RDS) [75]	3 items
	6	Sense of Collective Efficacy (SCE) [76]	Evaluates the effect of social and institutional mechanisms on people living in the neighborhood
	7	Neighborhood Disorder Scale (NDS) [75]	9 items
	8	Physical Conditions of the Neighborhood (PCN) [73]	7 items
	9	Facility in Neighborhood (FN) [77]	13 items; measures 3 domains: availability, utilization and quality
**Enabling factors**	10	Social Provisions Scale (SPS) [78]	Measures six items: emotional support, social integration, reassurance about his value, material help, counselling and information, need to feel useful
**Needs factors**	11	Composite International Diagnostic Interview (CIDI) and CIDI-SF [71,79]	Screening of mental disorders; was used in the World Mental Health 2000 (WMH 2000); included the most frequent mental disorders (mood disorders: depression, mania; anxiety disorders: social phobia, agoraphobia, and panic disorder, post-traumatic stress disorder (PTSD)). Screening of substance disorders (alcohol and drugs were based on the CIDI-Short Form (SF)
	12	Modified Observed Aggression Scale (MOAS) for aggressive behaviors [80]	Assess 4 categories of aggressive behavior: verbal aggression, aggression to propriety, self-inflicted aggression, physical aggression
	17	K-10 psychological distress scale (K-10 PDS) [81]	10 five-point Likert items; was used in the World Mental Health survey 2000 (WMH2000)
	18	Barratt Impulsivity Scale (BIS) [82]	30 four-point scale items organized in three categories: motor impulsivity, cognitive impulsivity, impulsivity due to lack of planning

### 2.4. Analyses

Analyses comprised univariate analyses, bivariate analyses, and multiple regression analyses. Univariate analyses entailed frequency distribution for categorical variables and mean values along with standard deviations for continuous variables. Bivariate analyses were carried out to assess variables significantly associated with healthcare service utilization for mental health reasons during the follow-up period, for an Alpha value of 0.010. Variables that passed this test were then used in a multiple regression model, for an Alpha value set at 0.05. Goodness-of-fit and total variance explained were generated. Comparison analyses were made on participant characteristics at T1 according to the presence of mental disorders at T1 and gender and healthcare service utilization for mental health reasons at T2.

## 3. Results and Discussion

### 3.1. Descriptive Analyses

The sample was described according to categorical variables (Table 2) and continuous variables (Table 3) at T1 for the 1823 remaining participants in the study at T2. There were twice as many females as males. The mean age was 43 years. Most participants had received education beyond secondary school level. The majority reported being dissatisfied or very dissatisfied with life and having a poor or fair perception of their physical and mental health. More than half attributed importance to spirituality. The most prevalent mental disorder was major depression.

### 3.2. Comparison Analyses according to the Presence of Mental Disorders and Gender Difference

Comparison analyses were made on participants’ general characteristics according to the presence of mental disorders and their gender at T1 and according to service utilization and gender at T2. As regards predisposing factors, being female, younger, having received less education, being dissatisfied with life, having a poor or fair perception of one’s physical and mental health, and problems with the law in the past 12 months and over a lifetime were found to be significantly associated with the presence of mental disorders. Enabling factors associated with the presence of mental disorders were unemployment, lower household and personal income, lower quality of life and physical conditions of the neighborhood, higher security, community involvement, sense of collective efficacy, resident disempowerment, and neighboring behavior scale score, and lower social support. Finally, needs factors associated with the presence of mental disorders were emotional problems, being a victim of violence, exhibiting aggressive behavior, and higher psychological distress score.

Concerning predisposing factors, females and males were significantly different with respect to self-perception of mental health (better for females), importance attributed to spirituality (more females than males), and problems with the law in the past 12 months and over a lifetime (mainly in males). Enabling factors were significantly different with respect to personal income (lower in females), security score (higher for females), community involvement score, sense of collective efficacy score, resident disempowerment score (lower for females), and social support score (higher for females).

**Table 2:** Participant characteristics by mental health status, gender and healthcare service utilization: categorical variables.

Factors	Variables	Categories	Participant Characteristics at T1								
			Total Sample	Mental Disorders		Gender		Healthcare Service Utilization at T2		Healthcare Services Users by Gender (*n* = 243)	
			n = 1823	No.	Yes	Female	Male	No	Yes	Female	Male
				n = 1580	n = 243	n = 1147	n = 676	n = 1581	n = 243	n = 159	n = 84
			%	%	%	%	%	%	%	%	%
**Predisposing factors**	Gender	Female	62.6	61.4	69.1 *			62.2	65.4		
		Male	37.4	38.6	30.9			37.8	34.6		
	Education	Secondary school or less	18.9	17.6	25.8 *	19.4	18.2	18.0	24.7 *	25.8	22.6
		Over secondary school	81.1	82.4	74.2	80.6	81.8	82.0	75.3	74.2	77.4
	Satisfaction with life	Satisfied or Very satisfied	82.3	87.2	56.7 **	81.7	83.4	85.7	60.5 **	61.6	58.3
		Neither satisfied nor dissatisfied	11.6	9.4	23.4	12.2	10.7	10.0	22.2	19.5	27.4
		Very dissatisfied or dissatisfied	6.0	3.4	19.9	6.1	5.9	4.3	17.3	18.9	14.3
	Self-perception of physical health	Excellent or very good	45.6	49.5	25.1 *	45.4	46.1	48.3	28.4 **	27.0	30.9
		Good	36.7	36.7	36.8	36.7	36.7	36.2	39.9	40.3	39.3
		Poor or fair	17.7	13.8	38.1	18.0	17.2	15.5	31.7	32.7	29.8
	Self-perception of mental health	Excellent or very good	58.6	65.5	22.3 **	55.9	63.1 *	63.4	27.2 **	26.4	28.6
		Good	30.6	28.3	42.3	32.1	27.9	29.0	40.7	39.6	42.9
		Poor or fair	10.9	6.2	35.4	12.0	9.0	7.6	32.1	34.0	28.6
	Importance attributed to spirituality		57.8	57.1	61.2	60.9	52.6 *	57.3	60.9	64.2	54.8
	Problems with the law in past 12 months		1.1	0.6	3.8 **	.4	2.2 **	1.0	1.6	0.0	4.8 *
	Lifelong history of problems with the law		5.4	3.8	13.7 **	2.8	9.7 **	4.3	12.3 **	6.9	22.6 *
**Enabling factors **	Source of income	From job	58.9	61.1	47.1 **	58.5	59.5	60.7	46.9 **	54.0	51.2
		Others	41.1	38.9	52.9	41.5	40.5	39.3	53.1	45.9	48.8
**Needs factors**	Mental disordersin previous 12 months	Major depression	8.6	0.0	54.0	9.6	6.9 *	5.4	29.2 **	28.9	29.8
		Mania	1.5	0.0	9.6	1.4	1.8	1.0	4.9 **	2.5	9.5 *
		Panic Disorder	1.8	0.0	11.3	2.4	0.9 *	1.3	4.9 **	5.7	3.6
		Social Phobia	3.3	0.0	21.0	4.4	1.6 *	2.3	10.3 **	12.6	5.9
		Agoraphobia	1.4	0.0	8.9	1.8	0.7 *	0.8	5.8 **	6.3	4.8
		Alcohol Dependence	2.7	0.0	17.2	1.9	4.1 *	1.4	11.5 **	8.2	17.9 *
		Drug Dependence	2.2	0.0	13.7	1.5	3.4 *	1.1	9.1 **	6.3	14.3 *
		PTSD	0.8	0.0	4.8	1.2	0 *	0.4	2.9 **	4.4	0.0 *
	Mental disorders in past 12 months (Yes/No)		16.0			17.6	13.2 *	10.9	49.0 **	30.8	20.2
	Emotional problems in past 12 months		33.2	28.9	55.7 **	36.9	27.0 **	31.4	44.9 **	50.3	34.5 *
	Victim of violence in past 12 months		5.1	4.0	11.0 **	5.0	5.3	4.3	10.3 **	8.2	14.3
	Aggressive behaviors in past 12 months		13.4	11.2	25.1 **	13.3	13.7	13.0	16.5	15.7	17.9

Notes: * *p* ≤ 0.5; ** *p* < 0.001.

**Table 3:** Participant characteristic by mental health status, gender and healthcare service utilization: continuous variables.

Factors	Participant Characteristics at T1																	
	Total Sample		Mental Disorders				Gender				Healthcare Service Utilization at T2				Healthcare Services Users by Gender *n* = 243			
	*n* = 1823		No		Yes		Female		Male		No		Yes		Female		Male	
			*n* = 1580		*n* = 243		*n* = 1147		*n* = 676		*n* = 1581		*n* = 243		*n* = 159		*n* = 84	
	Mean	SD	Mean	SD	Mean	SD	Mean	SD	Mean	SD	Mean	SD	Mean	SD	Mean	SD	Mean	SD
**Predisposing factors**																		
Age	42.6	13.2	42.9	13.3	41.0	12.5 *	42.7	13.1	42.3	13.3	42.7	13.2	41.5	12.9	42.5	3.0	39.6	12.5
Number of children in household	1.7	0.6	1.7	0.6	1.7	0.5	1.7	0.7	1.7	0.6	1.7	0.6	1.7	0.5	1.7	0.5	1.6	0.5
**Enabling factors**																		
Household income	60,803.5	49,127.4	63,746.2	50,791.2	45,311.4	35,462.8 **	59,848.4	48,790.5	62,405.1	49,682.2	62,832.3	50,392.2	47,612.0	37,424.9 **	48,708	39,282	45,538	33,759
Personal income	33879.1	31075.0	35515.8	32681.8	25262.8	18,425.6 **	30,996.0	23,895.7	38,713.9	39,898.7 **	34,974.5	32,182.1	26,756.7	21,305.6 **	26,027	20,441	28,137	22,915
Quality of life	109.4	15.9	111.9	14.3	96.5	17.4 **	109.8	15.6	108.9	16.3	111.2	14.7	97.8	17.9 **	98.6	17.9	96.3	18.0
Physical Conditions of the Neighborhood score	45.0	11.1	45.6	10.9	41.6	11.4 **	45.1	11.6	44.8	10.3	45.3	10.9	42.9	11.8 *	44.0	12.0	40.8 *	11.3
Security score	3.7	1.3	3.6	1.3	4.0	1.4 **	3.8	1.4	3.6	1.3 *	3.7	1.3	3.9	1.4 *	4.0	1.5	3.7	1.3
Community involvement scale score	9.1	1.1	9.1	1.1	9.2	1.0	9.1	1.1	9.2	1.0 *	9.1	1.1	9.3	1.0 *	9.2	1.1	9.4	1.0
Sense of collective efficacy score	26.4	6.0	26.2	5.9	27.5	6.5 **	26.0	6.0	27.0	5.9 *	26.2	5.9	27.6	6.4 *	26.9	6.5	29.0 *	6.1
Resident disempowerment scale score	11.5	6.0	11.2	5.9	12.9	6.4 **	11.1	6.1	12.0	5.9 *	11.3	6.0	12.5	6.2 *	22.1	6.4	13.3	5.8
Neighboring behavior scale score	14.4	8.5	14.2	8.4	15.4	9.0 *	14.7	8.6	14.0	8.3	14.3	8.5	15.1	8.4	15.3	8.4	14.9	8.3
Social support score	80.7	9.0	81.3	8.7	77.2	9.8 **	81.6	8.7	79.1	9.3 **	81.1	8.7	77.6	10.4 **	77.8	10.2	77.3	10.9
**Needs factors**																		
Number of mental disorders in past 12 months	0.2	0.6	0.0	0.0	1.4	0.7 **	0.2	0.6	0.2	0.6	0.1	0.4	0.8	1.0 **	1.0	0.0	1.0	0.0
Psychological distress score	8.2	6.5	6.8	5.3	15.4	7.6 **	8.5	6.8	7.5	6.0 *	7.2	5.7	14.2	8.0 **	13.4	7.9	11.7	8.3

Notes: * *p* ≤ 0.5; ** *p* < 0.001.

Concerning needs factors, they were significantly different as regards major depression, panic disorder, social phobia, agoraphobia, and PTSD (mostly in females), drug and alcohol dependence (mostly in males), mental disorders and emotional problems in the past 12 months (predominantly in females), and psychological distress score (higher for females).

Participants who used healthcare services for mental health reasons at T2 had the following characteristics at T1: lower education level and history of problems with the law (predisposing factors); unemployed, lower household and personal income, quality of life score, and physical conditions of the neighborhood score; higher security score, community involvement score, sense of collective efficacy score, and disempowerment score; and lower social support score (enabling factors); dissatisfied with their physical and mental health and their life, presence of mental disorders and emotional problems, and higher psychological distress score and number of mental disorders (needs factors). Females using healthcare services for mental health reasons were significantly less numerous than males in terms of having problems with the law both in the past 12 months and over their lifetime (predisposing factors) The physical conditions of the neighborhood score was significantly higher but their sense of collectivity efficacy score was significantly lower than for males (enabling factors). Finally, they were significantly less numerous than males to have mania and drug and alcohol dependence but significantly more numerous to have emotional problems (need factors).

### 3.3. Variables Associated with Healthcare Service Utilization for Mental Health Reasons

Variables significantly associated with healthcare service utilization for mental health reasons during the follow-up period in bivariate analyses were used to build the multiple logistic regression model presented in Table 4. As shown in this model, seven variables were found to be independently associated. Two of them were negatively associated: male gender and quality of life. Five others were positively associated: self-perception of physical health, major depressive episode, panic disorder, social phobia, and emotional problems, all in the past 12 months. This model yielded an acceptable goodness-of-fit and explained 66% of the total variance.

**Table 4:** Predictors of healthcare service utilization in the general population: multiple logistic regression (n = 1.823).

Predictors	Beta	SE	Wald	df	*p*	OR	95% CI	
							LL	UL
Gender (males)	−0.933	0.171	29.691	1	0.000	0.393	0.281	0.550
Self-perception of physical health	0.211	0.070	9.078	1	0.003	1.235	1.077	1.417
Major Depressive Episode in past 12 months	0.584	0.215	7.369	1	0.007	1.793	1.176	2.733
Panic Disorder in past 12 months	0.720	0.391	3.398	1	0.065	2.055	0.956	4.419
Social Phobia in past 12 months	0.844	0.297	8.104	1	0.004	2.326	1.301	4.159
Emotional problems in past 12 months	0.431	0.149	8.348	1	0.004	1.539	1.149	2.062
Quality of life score	−0.022	0.001	217.466	1	0.000	0.978	0.976	0.981

Notes: Goodness of fit: Hosmer-Lemeshow test: Khi-square = 3.522; *p* = 0.897; Total variance explained: Nagelkerke R^2^ = 65.5%.

### 3.4. Comparison Analyses between Participants with and without Mental Disorders

Table 5 displays comparison analyses between participants with mental disorders (n = 119) and without mental disorders (n = 124), among the subsample of 243 subjects who used healthcare services for mental health reasons at T2. Comparison characteristics were variables measured at T1. Among predisposing factors, six variables were found to be significantly discriminating between the two groups: participants without mental disorders having used services for mental health reasons were significantly better educated, reported higher quality of life score, had an excellent or very good self-perception of both physical and mental health, had fewer problems with the law in the past 12 months and, marginally, over their lifetime. Among enabling factors, 11 variables were significantly discriminating: participants without mental disorders with greater household income, greater social support, residing in neighborhoods with better physical and lower social cohesion, and earning a higher mean of household income before income tax. 

**Table 5:** Comparative analyses between participants with *vs.* without mental disorders using mental healthcare services (*n* = 243).

Factors	Variables	Categories	Total Sample	No Mental Disorders	Mental Disorders	*p* value
			(*n* = 243)	(*n* = 124)	(*n* = 119)	
			*n* (%)/(Mean (SD))	*n* (%)/(Mean (SD))	*n* (%)/(Mean (SD))	
**Predisposing factors**	Education (*n* (%))	Secondary or more	183 (75.3)	101 (81.5)	82 (68.9)	0.023 ^PCT^
	Quality of life (Mean (SD))		97.8 (17.9)	105.2 (15)	90 (17.5)	0.000 ^StT^
	Self-perception of physical health (*n* (%))	Excellent or very good	69 (28.4)	41 (33.1)	28 (23.5)	0.001 ^PCT^
		Good	97 (39.9)	57 (46)	40 (33.6)	
		Poor or Fair	77 (31.7)	26 (21)	51 (42.9)	
	Self-perception of mental health (*n* (%))	Excellent or very good	66 (27.2)	50 (40.3)	16 (13.4)	0.000 ^PCT^
		Good	99 (40.7)	52 (41.9)	47 (39.5)	
		Poor or Fair	78 (32.1)	22 (17.7)	56 (47.1)	
	Lifelong history of problems with the law (*n* (%))		30 (12.3)	7 (5.6)	23 (19.3)	0.001 ^PCT^
**Enabling factors**	Household income (Mean (SD))		47,612.0 (37,424.9)	54,305.3 (42,077.7)	40,637.5 (30,508.7)	0.004 ^StT^
	Social support score (Mean (SD))		77.6 (10.4)	80 (9.6)	75.2 (10.6)	0.001 ^StT^
	Environmental variables	Neighborhood physical status (Mean (SD))	42.9 (11.8)	45.3 (11.3)	40.3 (11.9)	0.027 ^StT^
		Participation in activities in the neighborhood (Mean (SD))	9.3 (1.0)	9.2 (1.2)	9.4 (0.9)	0.012 ^StT^
		Score of social cohesion: readiness to protect neighbor’s home when absent (Mean (SD))	15.1 (8.4)	14.6 (8)	15.7 (8.8)	0.000 ^StT^
		Driving distance to the neighborhood community health center (in meters) (Mean (SD))	2099.5 (1329.9)	2270 (1448.9)	1921.8 (1173.3)	0.041 ^StT^
		Proportion of immigrant population in the neighborhood (Mean (SD))	22.7 (9.8)	24.6 (10.3)	20.8 (9)	0.003
		Mean household income in the neighborhood before income tax (Mean (SD))	61,932.5 (23,475.9)	65,022.4 (25,139)	58,712.9 (21,237.4)	0.036^ StT^
**Needs**	Psychological distress score (Mean (SD))		14.2 (8.0)	10.4 (6.5)	18.3 (7.5)	0.000^ StT^
	Impulsiveness score (Mean (SD))		64.2 (12.6)	59.9 (10.3)	68.6 (13.2)	0.000^ StT^
	Emotional problems in the 12 past months (*n* (%))		109 (44.9)	41 (33.1)	68 (57.1)	0.000 ^PCT^
	Victim of violence in the 12 past months (*n* (%))		25 (10.3)	4 (3.2)	21 (17.6)	0.000 ^FET^
	Aggressive behaviors in the 12 past months (*n* (%))		40 (16.5)	9 (7.3)	31 (26.1)	0.000 ^PCT^
**Healthcare service professionals utilization**	Visited a psychiatrist in the past 12 months (*n* (%))		64 (26.3)	25 (20.2)	39 (32.8)	0.026 ^PCT^

Notes: ^**FET**^ Fisher Exact Test; **^PCT^** Pearson Chi-square test; **^StT^** Student t test.

Those with mental disorders were more likely to live in shorter walking and driving distance from community health centers. Neighborhoods with a higher proportion of immigrants and, marginally, lower proportion of working population aged 15 years or more, were less likely to be home to individuals with mental disorders using services for mental health reasons. All needs factors analyzed were associated with the presence of mental disorders: psychological distress, impulsiveness, emotional problems, being a victim of violence, and displaying aggressive behavior. Finally, participants with mental disorders were more likely to visit healthcare service professionals—especially psychiatrists and, marginally less so, psychologists—than those without mental disorders.

### 3.5. Discussion

#### 3.5.1. Predictors of Healthcare Service Utilization for Mental Health Reasons

The first purpose of this longitudinal study was to identify predictors of healthcare service utilization for mental health reasons in a population cohort. Using the Andersen behavioral model and a comprehensive set of variables influencing healthcare service utilization for mental health reasons, we found that two predisposing factors (gender and self-perception of physical health); one enabling variable (quality of life) and four need factors (major depressive episode, panic disorder, social phobia and emotional problems) predicted healthcare service utilization for mental health reasons. These findings are consistent with previous cross-sectional studies that have highlighted the predominance of need factors as determinants of healthcare service utilization for mental health reasons [16,65,83].

Previous studies have shown that females have a higher rate of healthcare service utilization for mental health reasons than males [84,85]. This difference has been explained by the social anchorage theory [86,87]. According to this theory, the gender difference in healthcare service utilization for mental health reasons may be explained either by the cultural values and expectations associated with a specific gender or by the specific roles endorsed by males and females. It has also been suggested that females have a greater tendency to confide in friends and family; this may explain the likelihood that females with mental disorders seek help as soon as they are diagnosed [88]. This study also confirms other research showing that females reported more mental disorders than males [89,90,91,92]. In fact, as shown in Table 2, males and females who used services for mental health reasons generally differed significantly in many ways. Males had significantly more mania, alcohol and drug dependence (need factors), problems with the law (predisposing factors) than females and lived in neighborhoods with worse physical conditions but with a greater sense of collective efficacy (enabling factors). In contrast, there were significantly more females than males with PTSD (needs factor). Furthermore, our study is the first that shows that social cohesion increases the likelihood of healthcare service utilization for mental health reasons, particularly among males. In line with the social anchorage theory, we can hypothesize that males with more severe mental disorders (such as mania) or social problems (for example, problems with the law) together with the pressure from their social network are more likely to seek help. Social cohesion probably acts as a kind of social support, and numerous studies indicate that patients with more social support tend to seek help to meet their need for care [93].

To our knowledge, ours is the first study to show clearly that positive self-rated physical health predicts healthcare service utilization for mental health reasons. There have been numerous indications that poor or fair self-perception of health resulted in increased healthcare service utilization in general [94,95,96]. Many studies have also shown correlations between poor self-rated health and physical health conditions [97,98,99,100], chronic diseases [101], frequent hospitalizations [102] and greater mortality [103,104,105,106]. However, few studies have assessed the influence of self-rated physical health on service utilization for mental health reasons [65,107].

With regard to needs factors, our results were also consistent with the literature concerning factors correlated with healthcare service utilization for mental health reasons. Previous studies have shown that patients with more mental disorders used more healthcare services [108,109]. One study on healthcare service utilization for mental health reasons in Canada and in the United States reported that among the needs factors, depression was the most significant and common predictor of overall use of services for mental health reasons [21]. Other studies showed that patients with depression used different types of health professionals. In Australia, it has been reported that 60% of patients with depression were seen in a general medical clinic, 21% were seen by a psychologist or other nonmedical therapist, and 29% were seen by a psychiatrist. Patients with depressive disorders were also most likely to be seen by both a psychiatrist and another mental health specialty provider, such as a nurse or social worker (44%), but 40% were seen only by a mental health specialty provider other than a psychiatrist [110]. One study involving 1572 Dutch subjects in an adult population with major or minor lifetime depression showed that 73% of subjects with depression had sought specialized mental healthcare or, to a lesser extent, primary care [111]. In another study, authors found that depressed elderly medical in-patients used more hospital and out-patient medical services than non-depressed patients [112].

Some authors have reported that patients with social phobia consult more specialist physicians [113] because their condition is either undiagnosed or deemed by physicians to be unlikely to benefit from early treatment designed to alter the course of the illness [114]. 

Although we found a correlation between panic disorder and healthcare service utilization for mental health reasons, one study conducted in the United States revealed that a minority of patients with this condition used healthcare services [61]. Another research drawn from the Healthcare for Communities study, a national household survey of the adult population in the United States, showed that panic disorder was also associated with a greater likelihood of healthcare service utilization, but not with the intensity of mental healthcare services. According to these authors, the elevated rate of healthcare service utilization among patients with panic disorder may be explained by the fact that panic disorder seems to be associated with increased odds of several comorbid disorders, including depression, dysthymia, psychosis, generalized anxiety disorder, bipolar disorder, and alcohol and drug use disorders [115].

Generally, cross-sectional and epidemiological studies have found healthcare service utilization to be associated with mental disorders, but not with emotional problems. In our study, emotional problems are not synonymous with psychological distress, which is usually reported to be strongly associated with healthcare service utilization for mental health reasons [116,117], or with poor self-perception of mental health. Some studies revealed that healthcare service utilization for mental health reasons among children with emotional problems begins at a very young age and occurs in multiple service sectors [118,119]. It is possible that individuals who experience emotional problems at a younger age are more likely to consult healthcare professionals as a preventive measure.

Finally, the only enabling factor that predicts healthcare service utilization for mental health reasons was low quality of life. In fact, according to numerous authors, low quality of life appeared to be a powerful indicator for healthcare service utilization for mental health reasons [120,121,122] and has been found to be a significant predictor of 30-day and one-year hospitalization [123].

#### 3.5.2. Correlates Associated with Healthcare Service Utilization for Mental Health Reasons among Individuals with and without Mental Disorders Respectively

The second purpose of this longitudinal study was to identify correlates associated with healthcare service utilization for mental health reasons among individuals with and without mental disorders respectively. The proportion of users without mental disorders (51%) in our study is similar to that found in previous studies [60,61,62,63,64]. Using the Andersen behavioral model, we found that differences among the two groups were mainly associated with enabling factors (n = 11), followed by predisposing factors (n = 6), needs factors (n = 5), and, finally, variables relating to healthcare service professionals (n = 4). The results show that healthcare service utilization for mental health reasons among individuals without mental disorders was strongly associated with enabling factors such as social support, income, environmental variables, and self-perception of the neighborhood.

Social support is acknowledged to be both a protecting factor against mental disorders and a strong predictor of healthcare service utilization for mental health reasons [124,125,126,127]. Spouses and relatives can help individuals recognize their problems and seek help from mental healthcare services [127]. Furthermore, since household and not personal income was associated with healthcare service utilization for mental health reasons among individuals without mental disorders, this may indicate that they don’t live alone. A higher income is associated with healthcare service utilization for mental health reasons mainly for services from psychologists, a class of professionals that is not usually covered by public healthcare systems [46,47]. In previous research undertaken in the same epidemiological catchment area, we found that among individuals with mental disorders, those with the highest household income at T1 also favored psychologists as the professionals most often consulted after general practitioners [16]. For individuals with low income, cost is the most important barrier to access to psychotherapy [128]. Moreover, individuals without mental disorders living in neighborhoods with better physical conditions and a higher mean household income before income tax perceived social cohesion and participation in activities in their neighborhood less favorably than individuals with mental disorders. This apparent contradiction may reflect greater individualism and loneliness among individuals without mental disorders [129,130]. In addition, according to the literature, social cohesion and social solidarity are weaker in neighborhoods with stronger ethnic diversity [131,132]. When the population is too heterogeneous, individuals are less likely to trust their neighbors [131]. In our study, neighborhoods with a higher proportion of immigrants were more often associated with individuals without mental disorders using services for mental health reasons. These conditions may be the source of emotional problems and stress among individuals without mental disorders and account for their healthcare service utilization for mental health reasons. Conversely, the fact that individuals with mental disorders using services for mental health reasons were more likely to live in neighborhoods with a lower proportion of immigrants makes sense. First, mental disorder is less prevalent among immigrants because potential new arrivals presenting with chronic disease are generally not admitted into the country [38]. Secondly, immigrants of the same ethnic group generally engage in mutual aid, which helps to protect them from mental disorders regardless of their socio-economic conditions [132,133]. Furthermore, it is logical to posit that individuals with mental disorders live closer to a community health center (offering primary healthcare and mental healthcare services), especially if they were more likely to be low-income earners with limited access to transportation. Individuals with mental disorders tend to live near their treatment center [65].

It was also expected that participants without mental disorders would be less likely to have need factors and visit professionals than individuals with mental disorders. Emotional problems were the most prevalent needs domains among individuals without mental disorders. As indicated previously, emotional problems are usually detected at a young age [118,119]. It is possible that some individuals without mental disorders consult professionals for mental health reasons over the long term for their emotional problems or to prevent relapses. Furthermore, the relatively high proportion of individuals without mental disorders (20%) who consulted a psychiatrist in the past 12 months seems to confirm the existence of previous diagnoses, including diagnoses of severe mental disorders or personality disorders that are not reported in the study.

### 3.6. Study Limitations 

Several limitations to the present study must be acknowledged. The first limitation stems from the sampling design, which enrolled subjects in a catchment area. This may limit the generalizability of the results. However, the high heterogeneity of the population within the catchment area may offset these limitations. The second limitation is that the Composite International Diagnostic Interview (CIDI) and the Composite International Diagnostic Interview Short Form (CIDI-SF) report only mood disorders, anxiety disorders, and drug and alcohol dependence; as a result, some mental disorders (for example, schizophrenia, personality disorders, and eating disorders) could not be included in the analysis. Previous studies have shown that patients with severe mental disorders, mainly schizophrenia, use more healthcare services than patients with disorders of moderate-to-low severity [134,135]. The third limitation is that we can make hypotheses only regarding the type of care used by participants. Even if females seem to be more likely to use healthcare services for mental health reasons, studies consistently show that men use more specialized care services than females [136,137]. Finally, the fourth limitation is that we did not have information concerning the frequency of visits to professionals. According to Druss *et al.*, a lower proportion of individuals without mental disorders use services [60].

## 4. Conclusions 

The strengths and originality of this study are found in its methodology: an epidemiological catchment area study was used, including a longitudinal survey (measured on two separate occasions), based on a comprehensive framework (Andersen model). There is a lack of literature on longitudinal predictors of healthcare service utilization for mental health reasons; as a result, the findings of the present study are of great significance. In addition, better knowledge of factors distinguishing users of healthcare service utilization for mental health reasons with and without mental disorders would be useful in improving the distribution of healthcare services.

Consistent with past cross-sectional research, the study showed that all three components of the behavioral model contributed to healthcare service utilization for mental health reasons and that needs factors (namely, mental disorders and emotional problems) were the major predictors. Males also appear to be less likely to seek care until their illness or perceived health concern is more severe. This may increase the risk of crisis and/or suicide. Consequently, outreach initiatives should aim to improve service utilization among males. Two other predictors of healthcare service utilization for mental health reasons were also uncovered: emotional problems and good self-rated physical health. These results suggest that general practitioners, in their role as the point of entry into the healthcare system (as well as access to other primary care providers), should be more sensitive to these variables in routine primary care if they are to prevent and treat mental disorders more effectively. Accordingly, healthcare service utilization for mental health reasons may be fostered through more effective screening of patients entering the system with less than optimal self-perception of physical health or other emotional and quality of life issues.

Furthermore, our study results show that enabling factors, mainly geographical variables and self-perception of the neighborhood, are key to distinguishing users of healthcare services for mental health reasons among individuals with and without mental disorders, respectively. More specifically, our study found that lower social cohesion and social solidarity in neighborhood settings are two original variables that contribute to healthcare service utilization for mental health reasons among individuals without mental disorders. These results confirm the importance for mental healthcare services to take neighborhoods into account and adjust care provision accordingly. Initiatives that facilitate the integration of immigrants and participation in activities in neighborhood settings may reduce the need, among individuals without mental disorders, to seek help. Finally, as our results indicate, we need to understand why individuals without a mental disorder consult psychiatrists. Accordingly, more concerted efforts to direct these individuals to appropriate primary-care services may be needed.
